# The Timing of Surgery Following Stereotactic Body Radiation Therapy Impacts Local Control for Borderline Resectable or Locally Advanced Pancreatic Cancer

**DOI:** 10.3390/cancers15041252

**Published:** 2023-02-16

**Authors:** Timothy Lin, Abhinav Reddy, Colin Hill, Shuchi Sehgal, Jin He, Lei Zheng, Joseph Herman, Jeffrey Meyer, Amol Narang

**Affiliations:** 1 Department of Radiation Oncology & Molecular Radiation Sciences, Johns Hopkins University School of Medicine, Baltimore, MD 21287, USA; 2 Department of Radiation Oncology, New York University Langone Health, New York, NY 10016, USA; 3 Department of Surgery, Johns Hopkins University School of Medicine, Baltimore, MD 21287, USA; 4 Department of Medical Oncology, Johns Hopkins University School of Medicine, Baltimore, MD 21287, USA; 5 Department of Radiation Oncology, Northwell Health Cancer Institute, New Hyde Park, NY 11042, USA

**Keywords:** pancreatic adenocarcinoma, timing of surgery, stereotactic body radiation therapy

## Abstract

**Simple Summary:**

The role of radiation therapy in localized pancreatic cancer is controversial. The interval of time from the end of radiotherapy to surgery has been shown in some cancer disease sites to affect subsequent outcomes but has not been studied in pancreatic cancer. We aimed to characterize the optimal timing of surgery following stereotactic body radiation therapy (SBRT) in patients with borderline resectable or locally advanced pancreatic cancer. We found that patients who underwent surgery more than 6 weeks after completing SBRT had improved local control outcomes compared to those who had received surgery within 6 weeks of completing SBRT, even when controlling for other clinical factors such as the pathological response to neoadjuvant chemotherapy. These findings could be used to inform future studies, including prospective trials to better select patients with localized pancreatic cancer who may benefit from radiotherapy.

**Abstract:**

We aimed to evaluate the impact of time from stereotactic body radiation therapy (SBRT) to surgery on treatment outcomes and post-operative complications in patients with borderline resectable or locally advanced pancreatic cancer (BRPC/LAPC). We conducted a single-institutional retrospective analysis of patients with BRPC/LAPC treated from 2016 to 2021 with neoadjuvant chemotherapy followed by SBRT and surgical resection. Covariates were stratified by time from SBRT to surgery. A Cox regression model was used to identify variables associated with survival outcomes. In 171 patients with BRPC/LAPC, the median time from SBRT to surgery was 6.4 (range: 2.7–25.3) weeks. Hence, patients were stratified by the timing of surgery: ≥6 and <6 weeks after SBRT. In univariable Cox regression, surgery ≥6 weeks was associated with improved local control (LC, HR 0.55, 95% CI 0.30–0.98; *p* = 0.042), pathologic node positivity, elevated baseline CA19-9, and inferior LC if of the male sex. In multivariable analysis, surgery ≥6 weeks (*p* = 0.013; HR 0.46, 95%CI 0.25–0.85), node positivity (*p* = 0.019; HR 2.09, 95% CI 1.13–3.88), and baseline elevated CA19-9 (*p* = 0.002; HR 2.73, 95% CI 1.44–5.18) remained independently associated with LC. Clavien–Dindo Grade ≥3B complications occurred in 4/63 (6.3%) vs. 5/99 (5.5%) patients undergoing surgery <6 weeks and ≥6 weeks after SBRT (*p* = 0.7). In summary, the timing of surgery ≥6 weeks after SBRT was associated with improved local control and low post-operative complication rates, irrespective of the surgical timing. Further investigation of the influence of surgical timing following radiotherapy is warranted.

## 1. Introduction

Pancreatic cancer is the third most common cause of cancer death in the United States [1], despite being the 10th most common cancer by incidence [2]. In patients with localized disease, margin-negative surgical resection has been associated with improved outcomes [3]. Unfortunately, approximately half of patients have distant metastatic disease at the time of diagnosis [2]. Furthermore, patients with borderline resectable or locally advanced pancreatic cancer (BRPC/LAPC) are at high risk of R1 resection with upfront surgery due to the involvement of key peri-pancreatic vasculature.

The role of radiotherapy for localized pancreatic ductal adenocarcinomas is controversial [4,5]. In the LAPC setting, consolidative radiotherapy has historically been delivered with the intent of improving local control and preventing morbidity and mortality related to complications of local progression [6,7]. More recently, in the setting of modern chemotherapy and radiotherapy, it has been realized that a higher proportion of patients with LAPC may undergo complete resection following neoadjuvant therapy [8,9]. Radiotherapy in this context can therefore be delivered for margin sterilization and local recurrence risk reduction, similar to its intent in the setting of borderline resectable disease. However, recent trials examining radiotherapy for localized disease have yielded conflicting results. The PREOPANC study demonstrated a local recurrence and overall survival benefit to the addition of chemoradiotherapy to surgery in patients with resectable or borderline resectable disease [4]. However, the Alliance A021501 study showed no benefit to adding neoadjuvant stereotactic body radiation therapy (SBRT) to chemotherapy in the setting of BRPC [5].

Radiotherapy delivery parameters vary greatly across institutions and trials. To identify factors that may help to explain conflicting results seen in prospective and retrospective data, a closer examination of radiation therapy parameters may prove useful. One such parameter is the time from radiotherapy to surgical resection.

Surgical resection following neoadjuvant radiation commonly takes place 4–8 weeks following the completion of radiation; however, the importance of the timing of surgical resection following neoadjuvant radiotherapy and the ideal time at which resection should take place after radiation have not been explored in BRPC/LAPC. Studies in other malignancies, such as locally advanced rectal cancer patients, suggest that the timing of surgical resection with respect to delivery of radiation therapy may have implications with respect to tumor response, post-operative complications, and outcomes [10,11]. Thus, we aimed to characterize the impact of surgery following neoadjuvant radiation in borderline resectable and locally advanced pancreatic cancer on tumor response, surgical margins, post-operative complications, and survival outcomes. As our institution has commonly administered SBRT for localized PDAC, we specifically explore this in the setting of SBRT.

## 2. Materials and Methods

We retrospectively reviewed a cohort of patients with BRPC/LAPC treated at our institution with neoadjuvant chemotherapy consisting of modified FOLFIRINOX (mFOLFIRINOX) or gemcitabine/nab-paclitaxel, followed by SBRT to a total dose of 33 Gy in 5 fractions, and then surgical resection. A complete description of practice patterns at our institution has previously bene provided [12]. Briefly, upfront chemotherapy was first delivered, with the choice of regimen and duration at the discretion of the treating medical oncologist, but typically consisting of mFOLFIRINOX or gemcitabine/nab-paclitaxel over 4 months. Restaging computed tomography (CT) scans were obtained at 2-month intervals to assess treatment response. After the completion of chemotherapy, patients were typically recommended to undergo neoadjuvant SBRT. To allow for image guidance, endoscopic ultrasound-guided placement of fiducial markers was first performed, followed by a contrasted CT simulation scan. Patients were simulated in the supine position, with Vac-Lok or Alpha-Cradle used for immobilization. Motion management was typically achieved with active breathing coordination (ABC), though a few patients who could not tolerate ABC underwent free-breathing scans. Following simulation, the clinical target volume (CTV), consisting of all gross disease in addition to the full circumference of the involved vasculature at involved axial levels, was contoured. An internal target volume (ITV) was similarly delineated for patients with free breathing scans. A 2 mm isotropic expansion was applied to the CTV or ITV to obtain the planning target volume (PTV). The PTV was prescribed at 33 Gy in 5 daily fractions. Both pre-treatment and intra-fraction cone-beam CT-based image guidance were used, with alignment to the spine followed by a shift to the fiducial markers. Approximately 2–6 weeks following the completion of SBRT, the final determination of surgical resectability was made with a restaging CT scan. Following resection, adjuvant chemotherapy was delivered as per the medical oncologist’s discretion. After surgery, patients were followed with serial CT surveillance scans, initially at 3-month intervals, with the subsequent surveillance frequency determined by the multidisciplinary team.

Baseline demographics and clinical characteristics were collected from the electronic medical record for each patient. Surgical complications were determined using the Clavien–Dindo classification scheme, with grade 3B complications defined as those requiring surgical, radiologic, or endoscopic intervention under general anesthesia [13]. These covariates were then stratified by the timing of surgery following SBRT. The time from SBRT to surgery was calculated from the final day of SBRT to the day of surgery. Patients were stratified for analysis by the timing of surgery following SBRT, selecting a clinically pragmatic threshold based on the median timing in the cohort. All survival endpoints were defined from the completion of SBRT to the endpoint in question. The endpoint for local control (LC) was time to radiographic local recurrence in the post-operative bed as assessed by CT; similarly, the event of interest for freedom from distant metastases (FDM) was time to distant progression, while the event of interest for overall survival (OS) was death.

Pearson’s chi-squared test was used to compare baseline and tumor response characteristics according to the timing of surgery. Kaplan–Meier survival curves were generated for LC, FDM, and OS, stratified by the timing of surgery following SBRT. A Cox proportional hazards regression was performed to identify covariates associated with survival outcomes, with a *p*-value of less than 0.05 as the threshold for statistical significance. Covariates that were statistically significant in the univariable analysis were included in multivariable modeling.

## 3. Results

**Figure 1:**
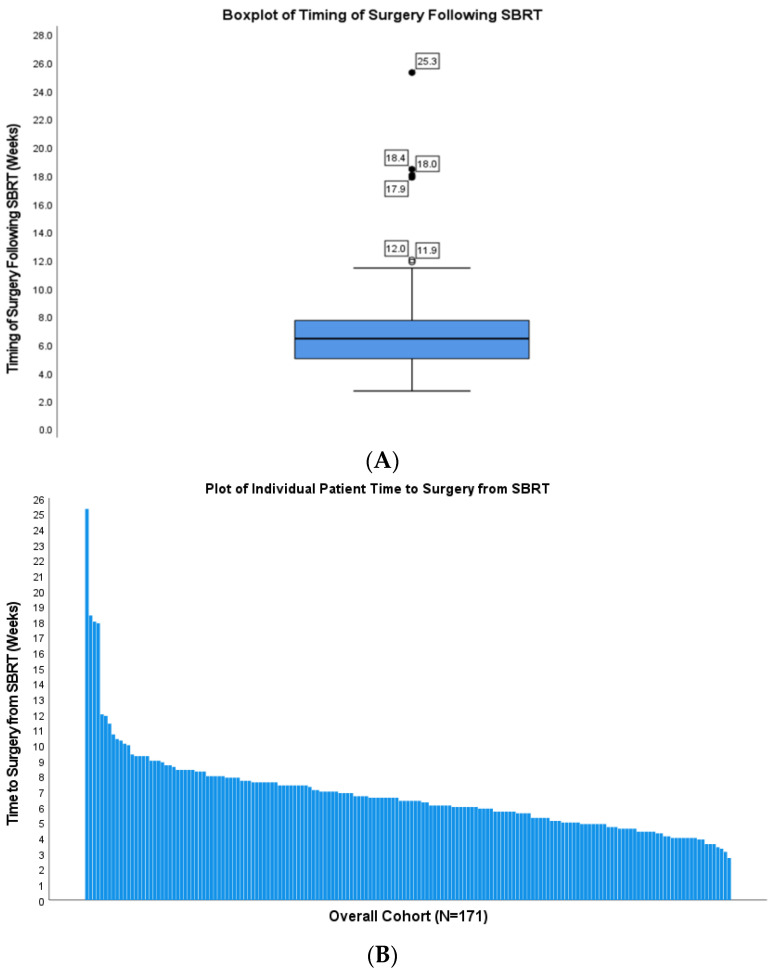
Boxplot (**A**) and waterfall plot (**B**) of the timing of surgery following SBRT for the overall cohort (N = 171). Abbreviations: SBRT, stereotactic body radiation therapy. (**A**) Numbers beside circles represent the time interval in weeks for individual patients from the completion of SBRT to surgical resection. (**B**) Individual bars represent individual patient intervals between the completion of SBRT and the time of surgical resection.

A total of 171 patients treated at our institution from 2016 to 2021 were included in the analysis. The median (IQR) follow-up was 13.9 (8.1–20.1) months. The median timing of surgery following SBRT was 6.4 weeks (range: 2.7–25.3, IQR 2.7 weeks; Figure 1) in the overall cohort. On this basis, a threshold of 6 weeks was chosen for assessing the differential impact of the timing of surgery following SBRT. In the overall cohort, the median age was 65.9 years; all patients received multi-agent chemotherapy consisting of mFOLFIRINOX or gemcitabine/nab-paclitaxel. The median duration of neoadjuvant chemotherapy was 4 months (IQR: 4–6 months). All patients were treated with SBRT of 33 Gy in 5 fractions. 

There was no statistically significant difference in the distribution of age, sex, tumor location, neoadjuvant chemotherapy type or duration, pathologic response, surgical margin status, pathologic node status, or receipt of adjuvant chemotherapy between patients who underwent surgery before or after 6 weeks from SBRT completion (Table 1). 

**Figure 2:**
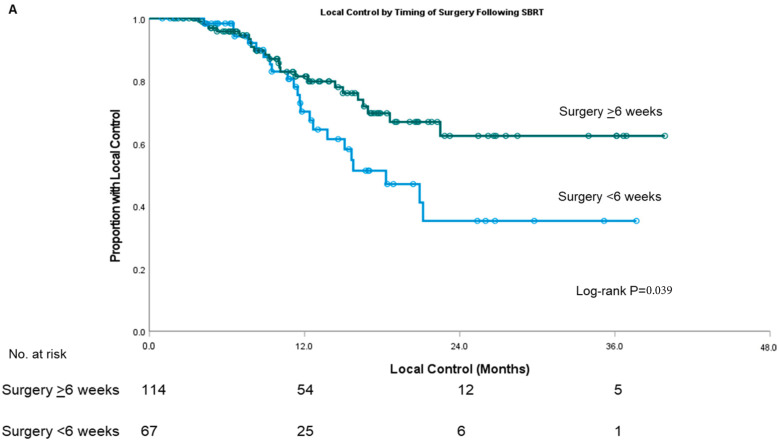

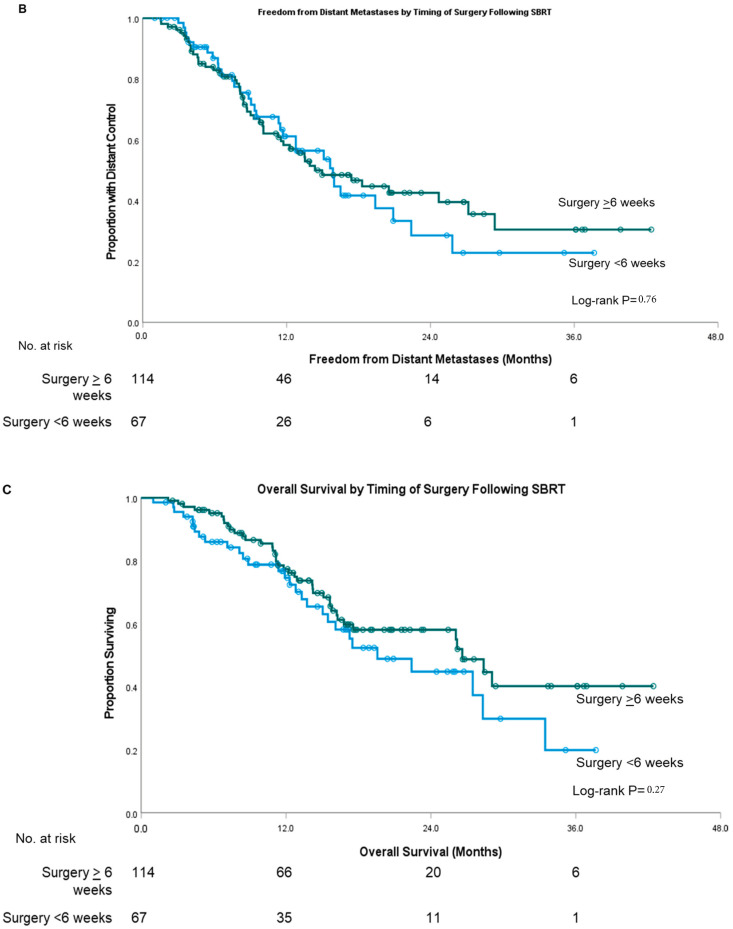
Kaplan–Meier survival curves for local control (**A**), freedom from distant metastases (**B**), and overall survival (**C**) stratified by the timing of surgery following SBRT. Time-to-event curves of patients who underwent surgery ≥6 weeks (green) vs. <6 weeks (blue) following the completion of SBRT. Circles indicate censored observations.

The median (IQR) follow-up was 14.8 (8.8–20.8) months in the surgery ≥6 weeks cohort and 12.3 (6.3–18.9) months in the surgery <6 weeks cohort. Local control was improved in those undergoing surgery ≥6 weeks following SBRT (1-year LC 81.5% vs. 70.2%; median LC not reached vs. 18.3 months; log-rank *p* = 0.039; Figure 2A). There was no significant difference in freedom from distant metastases (1-year FDM 58.3% vs. 61.1%; median FDM 14.4 vs. 15.9 months; *p* = 0.76; Figure 2B) or overall survival (1-year OS 77.4% vs. 74.6%; median OS 26.6 vs. 19.6 months; *p* = 0.27; Figure 2C) in those undergoing surgery ≥6 weeks following SBRT compared to <6 weeks. 

In univariable Cox regression (Table 2), there was no association between LC and age, neoadjuvant chemotherapy regimen or duration, tumor anatomic location, pathologic response, or surgical margin status. 

Time to surgery ≥6 weeks was associated with improved LC (*p* = 0.042; HR 0.55, 95% CI 0.30–0.98) but not associated with FDM (HR 0.93, 95% CI 0.60–1.45; *p* = 0.76; Supplemental Table S1) or OS (HR 0.74, 95% CI 0.46–1.19; *p* = 0.21; Supplemental Table S1). Pathological node-positive disease (*p* = 0.021; HR 2.01, 95% CI 1.11–3.63), baseline CA19-9 above 200 U/mL (*p* = 0.018; HR 2.09, 95% CI 1.14–3.84), and being of the male sex (*p* = 0.026; HR 2.02, 95% CI 1.09–3.75) were significantly associated with worse LC. 

In the multivariable analysis (Table 2), the timing of surgery following SBRT (*p* = 0.013; HR 0.46, 95% CI 0.25–0.85), pathologic node-positivity (*p* = 0.019; HR 2.09, 95% CI 1.13–3.88), and baseline elevated CA19-9 above 200 U/mL (*p* = 0.002; HR 2.73, 95% CI 1.44–5.18) remained significantly associated with LC, while being of the male sex (*p* = 0.06; HR 1.83, 95% CI 0.97–3.43) did not maintain significance. To better characterize the association between being of the male sex and worse prognostic outcomes, baseline characteristics were compared by sex, with higher rates of nodal positivity observed among male patients as compared to female patients (53% vs. 63%; *p* = 0.059). A second multivariable Cox regression was run as a sensitivity analysis, including clinically relevant covariates that did not meet initial statistical significance in univariable analysis, including surgical margin status, presence of pathologic complete response, disease stage at diagnosis (i.e., BR vs. LAPC), baseline performance status, and receipt of adjuvant chemotherapy, again showing preservation of the independent association of the three aforementioned significant covariates, with all other factors remaining non-significant.

An additional sensitivity analysis (Supplemental Table S2) was performed that excluded patients with pronounced delays (≥15 weeks) in surgical resection following SBRT (n = 4). Two of these patients had surgeries that were initially aborted due to initial concern for metastatic disease in the liver but subsequently underwent surgical resection. Of these patients, one initially had concerning findings on the frozen section, which were later found to represent a benign pathology. The other patient was found to have a biopsy-proven liver lesion, received further chemotherapy, and the lesion was eventually explored again and ultimately resected, with a wedge section of the initially involved site of disease in the liver returning no evidence of malignancy. One patient had attempted to enroll in a clinical trial but ultimately opted for surgical resection, resulting in a delay in surgery. For the final patient, it was initially unclear prior to SBRT whether the patient was a candidate for resection; however, following restaging post-SBRT, the surgical team determined the patient was a candidate for resection, and the patient remained on neoadjuvant chemotherapy until successful resolution of care coordination challenges, after which time the patient underwent resection. After the exclusion of these 4 patients, LC remained significantly improved in patients with surgery ≥6 weeks following SBRT (*p* = 0.037; HR 0.53, 95% CI 0.29–0.96), while there remained no significant difference in FDM and OS. Pathologic node positivity, baseline CA19-9, and sex similarly remained significant in the univariable analysis. Time from SBRT to surgery ≥6 weeks, pathologic node positivity, elevated baseline CA19-9, and sex all maintained significance in the multivariable analysis of LC (Supplemental Table S2).

There was no significant difference in the frequency of significant Clavien–Dindo (i.e., Grade 3B or higher) complications between the cohort that underwent surgery prior to 6 weeks (4/63 patients, 6.3%) and to the cohort after 6 weeks (5/99 patients, 5.5%; *p* = 0.7). Complications in those who underwent surgery >6 weeks post-SBRT were as follows: bleeding from the common hepatic artery in the region of the gastroduodenal artery (GDA) stump, requiring GDA stump coiling; pseudoaneurysm of the GDA, requiring embolization, approximately 2 years after initial resection; abscess of the splenectomy bed, approximately 2 weeks post-operatively, requiring drain placement by interventional radiology; gastrojejunal perforation, approximately 2 years post-resection, requiring exploratory laparotomy with the repair of the perforation; and purulent sinus tract drainage to the skin, approximately 1 month post-operatively, requiring endoscopic intervention. Complications for those undergoing surgery <6 weeks following SBRT were biliary stricture at the hepaticojejunostomy site requiring percutaneous biliary drain placement; gastrointestinal bleed requiring upper esophagogastroduodenoscopy; GI bleed with pseudoaneurysm of the GDA stump requiring embolization, approximately 2 years post-resection; and GI bleed from the GDA stump requiring coil embolization, approximately 4 months post-resection.

## 4. Discussion

The optimal timing of surgery following SBRT in patients with BRPC/LAPC who receive neoadjuvant chemotherapy is not known. This hypothesis-generating study suggests that surgery performed over 6 weeks after the completion of SBRT may be associated with improved local control compared to surgery within an earlier timeframe, independent of other prognostic factors including baseline CA19-9 levels and pathologic nodal status. This finding, even in the absence of improved overall survival, is of clinical significance to patients with pancreatic cancer, in whom a significant portion of patients die of local tumor invasion even after experiencing distant failure [6]. Furthermore, as systemic therapy options aimed at improving metastatic pancreatic cancer continue to be developed, the clinical relevance of local control will likely continue to grow.

The timing of surgery following radiotherapy has varied across notable pancreatic cancer trials. In the Alliance A012501 trial, surgery was mandated within 4–8 weeks of radiotherapy, resulting in a median time to surgery of 44 days (range 26–62 days) in the radiotherapy arm [5]. Similarly, in A021101, in which patients received preoperative mFOLFIRINOX followed by chemoradiotherapy, surgery was mandated within 4–10 weeks of radiotherapy, and the median time to surgery was 6.3 weeks (range 4.1–11.1 weeks) [14]. In the PREOPANC study, surgery was to take place 14–18 weeks after randomization, while chemoradiotherapy, consisting of 3 cycles of gemcitabine every 4 weeks with a 3-week radiotherapy course (36 Gy in 15 fractions) delivered concurrently with cycle 2, was to begin within 4 weeks of randomization. While the timing results were not reported, based on the protocol, surgery would have been mandated within 3–11 weeks following the completion of radiotherapy [15]. Future prospective randomized trials in borderline resectable or locally advanced pancreatic cancer studies would benefit from greater consideration of the potential significance of the time interval to surgical resection following neoadjuvant therapy during trial design. In the meantime, in the absence of prospective data to guide recommendations on the timing of surgery, an individualized, multidisciplinary discussion weighing the risks and benefits for each patient is required to determine the timing of surgery following radiotherapy.

The mechanism through which increased time from radiation to surgery may improve local control is unclear from our data. Interestingly, in the later surgical timing cohort, we did not observe improved rates of margin-negative resection, which has been correlated with improved outcomes in some studies [3,16,17], with less clear associations in others [18,19]. The lack of association between margin status and outcomes in our study as in others may reflect inherent limitations and variability in definitions of margin status [20,21,22] across studies of patients with pancreatic cancer, or perhaps variability in surgical technique or the need for vascular resection. Additionally, while there is radiobiological plausibility to support the hypothesis that an increased window of time between SBRT and surgery may facilitate higher rates of margin-negative resection, studies of the timing of pre-operative radiotherapy in locally advanced rectal cancer have not consistently demonstrated this relationship either [10,23].

In our study, there were also no differences in rates of pathologic complete response or post-operative complication rates according to the timing of surgery following SBRT. Extrapolating from trials in locally advanced rectal cancer literature, the impact of delaying surgery following radiotherapy on pathologic response rates is unclear, with evidence both in support of [11] and in contradiction to [10] the hypothesis that delaying surgery improves pathologic complete response rates. Available data from rectal cancer trials also suggest increasing the time from the end of radiotherapy to surgery may negatively impact morbidity and the surgical quality of resection [10], a metric that has been linked to outcomes [24], perhaps owing to increased fibrosis from radiotherapy with delayed surgery, which leads to a more challenging dissection. In our study, significant post-operative complication rates were infrequent, independent of the timing of surgery. This finding may in part reflect treatment at a high-volume center with significant experience in performing oncologic resections in BRPC/LAPC patients following neoadjuvant chemotherapy and SBRT, given the positive association that has been reported between surgical volume and subsequent outcomes [25]. To our knowledge, a standardized assessment of surgical quality in pancreatic cancer that is analogous to TME quality assessment in rectal cancer [26] does not currently exist, which in part may relate to variability in the determination of margin status, as previously described [27]. Without a more detailed recording of surgical outcomes, including the extent of resection and characterization of the degree of fibrosis intra-operatively, it will be challenging to determine the relationship between the timing of surgery and subsequent local control. Future studies of the timing of surgery after neoadjuvant SBRT in pancreatic cancer may benefit from a more formalized assessment of surgical parameters related to the presence of fibrosis and quality of dissection, which can then be used as an additional point of comparison between cohorts.

Increasing the interval from the completion of radiation therapy to surgical resection in BRPC/LAPC increases the time off of full-dose chemotherapy. One benefit of SBRT is that it lessens the need for breaks from systemic therapy, as is needed with conventionally fractionated therapy, which typically employs sensitizing rather than full-dose chemotherapy. A future consideration would be to explore the potential impact of interdigitating SBRT between chemotherapy cycles to facilitate earlier delivery in the neoadjuvant course, as some have proposed [28].

Our study is inherently limited by its retrospective design and is therefore primarily useful for hypothesis generation. Another potential limitation is selection bias in the delayed surgery cohort, given that patients in this cohort had a longer period of time during which progressive disease could declare itself prior to surgery, potentially enriching the cohort with patients with inherently favorable tumor biology. However, patients in both cohorts were treated with similar neoadjuvant chemotherapy regimens with similar durations, and to receive those, they were already selected for having a favorable disease. Furthermore, surgery in this study primarily took place in the 4–8-week window during which it seems less plausible that drastic differences in progression related to a pause in systemic therapy would be observed. To further mitigate the possibility that favorable selection in the delayed surgery cohort could account for differential outcomes in this study, we conducted a sensitivity analysis excluding four patients with the longest intervals between SBRT and surgery, ≥15 weeks, leading to confirmation of the results of the initial analysis.

## 5. Conclusions

Time to surgery greater than 6 weeks following the completion of neoadjuvant SBRT in patients with BRPC/LAPC was independently associated with improved local control even in the absence of detectable differences in the rates of pathologic response or R0 resection. Post-operative complication rates were low, irrespective of the surgical timing. Further investigation of the potential impact of the timing of neoadjuvant SBRT in pancreatic cancer management is an area of unmet need. Similar questions regarding the timing of SBRT in relation to chemotherapy have been investigated in the neoadjuvant management of rectal cancer and deserve further consideration in pancreatic cancer management.

## Figures and Tables

**Table 1:** Univariable and multivariable Cox proportional hazards regression for local control by the timing of surgery following SBRT.

Covariate		Surgery <6 Weeks from SBRT	Surgery ≥6 Weeks from SBRT	*p*-Value
**Number of Patients**		67	104	--
**Age Median (IQR)**		66.3	65.5	
**Sex**	Female	31 (46%)	57 (55%)	0.28
	Male	36 (54%)	47 (45%)	
**Race**	White	53 (79%)	90 (87%)	
	Other ^a^	14 (21%)	14 (13%)	
**Tumor Location**	Pancreas Head	40 (60%)	66 (63%)	0.62
	Other ^b^	27 (40%)	38 (37%)	
**Disease Stage at Diagnosis**	Borderline Resectable	34 (51%)	46 (44%)	0.40
	Locally Advanced	33 (49%)	58 (56%)	
**Surgery**	Whipple	46 (69%)	70 (67%)	0.51
	Total Pancreatectomy	1 (1%)	5 (5%)	
	Distal Pancreatectomy	20 (30%)	29 (28%)	
**Pathologic Complete Response**	No	63 (94%)	99 (95%)	0.74
	Yes	4 (6%)	5 (5%)	
**Surgical Margin**	R0	60 (90%)	95 (91%)	0.69
	R1 or Higher	7 (10%)	9 (9%)	
**Pathologic Nodal Status**	Negative	33 (49%)	60 (58%)	0.28
	Positive	34 (51%)	44 (42%)	
**Baseline ECOG**	0–1	65 (98%)	97 (96%)	0.37
	2 or Higher	1 (2%)	4 (4%)	
**Neoadjuvant Chemotherapy Duration**	≥4 Months	10 (15%)	10 (10%)	0.97
	<4 Months	57 (85%)	94 (90%)	
**Baseline CA19-9**	<200	35 (54%)	48 (48%)	0.43
	≥200	30 (46%)	53 (52%)	
**Adjuvant Chemotherapy**	No	39 (42%)	28 (36%)	0.53
	Yes	55 (58%)	49 (64%)	

Abbreviations: *p*, *p*-value; HR, hazard ratio; CI, confidence interval; SBRT, stereotactic body radiation therapy; ECOG, Eastern Cooperative Oncology Group; PS, performance status; FOLFIRINOX, folinic acid, fluorouracil, irinotecan, and oxaliplatin. ^a^ Other race categories included Asian and Black. ^b^ Other tumor locations included the pancreatic neck, body, tail, and uncinate process.

**Table 2:** Univariable and multivariable Cox proportional hazards regression for local control by the timing of surgery following SBRT.

Covariate		Univariable Cox *p*	Univariable Cox HR (95% CI)	Multivariable Cox *p*	Multivariable Cox HR (95% CI)
**Timing of Surgery Post-SBRT**	≥6 weeks	0.042	0.55 (0.30–0.98)	0.013	0.46 (0.25–0.85)
	<6 weeks		Reference		
**Pathologic Node Status**	Positive	0.021	2.01 (1.11–3.63)	0.019	2.09 (1.13–3.88)
	Negative		Reference		
**Baseline CA19-9, U/mL**	≥200	0.018	2.09 (1.14–3.84)	0.002	2.73 (1.44–5.18)
	<200		Reference		
**Age**		0.43	0.99 (0.95–1.02)		
**Sex**	Male	0.026	2.02 (1.09–3.75)	0.06	1.83 (0.97–3.43)
	Female	--	Reference		
**ECOG PS**	2 or Higher	0.77	1.35 (0.19–9.80)		
	0–1		Reference		
**Tumor Location**	Other ^a^	0.44	0.78 (0.42–1.46)		
	Head		Reference		
**Neoadjuvant Chemotherapy Regimen**	FOLFIRINOX	0.86	1.07 (0.50–2.31)		
	Gemcitabine/Nab-paclitaxel		Reference		
**Neoadjuvant Chemotherapy Duration**	≥4 Months	0.26	0.63 (0.28–1.40)		
	<4 Months				
**Pathologic Complete Response**	Present	0.18	0.26 (0.04–1.86)		
	Absent		Reference		
**Surgical Margin**	R0	0.40	0.45 (0.61–3.43)		
	R1 or Higher		Reference		
**Adjuvant Chemotherapy**	Yes	0.38	0.81 (0.51–1.30)		
	No		Reference		

Abbreviations: *p*, *p*-value; HR, hazard ratio; CI, confidence interval; SBRT, stereotactic body radiation therapy; ECOG, Eastern Cooperative Oncology Group; PS, performance status; FOLFIRINOX, folinic acid, fluorouracil, irinotecan, and oxaliplatin. ^a^ Other tumor locations included pancreatic neck, body, tail, and uncinate process.

## Data Availability

The data are not publicly available due to the presence of protected health information.

## Supplementary Materials

The following supporting information can be downloaded at: https://www.mdpi.com/article/10.3390/cancers15041252/s1, Table S1: Univariable and multivariable Cox proportional hazards regression for freedom from distant metastases and overall survival by the timing of surgery following SBRT in the overall cohort; Table S2: Sensitivity analysis excluding 4 patients who received surgery ≥15 weeks after the completion of SBRT.
