# Supplementary material for: The Timing of Surgery Following Stereotactic Body Radiation Therapy Impacts Local Control for Borderline Resectable or Locally Advanced Pancreatic Cancer
Source: Cancers (Basel). 2023 Feb 16;15(4):1252. doi: 10.3390/cancers15041252 (PMC9954439; doi:10.3390/cancers15041252)
Supplement: Supplementary file 1 [file cancers-15-01252-s001.zip › cancers-2207531-supplementary.pdf]

Supplemental Table S1. Univariable and multivariable Cox proportional hazards regression for freedom from distant metastases and overall survival by timing of surgery following SBRT in the overall cohort (N=171).

Freedom from distant metastases

Covariate		Univariable Cox P	Univariable Cox HR (95% CI)	Multivariable Cox P	Multivariable Cox HR (95% CI)
**Timing of Surgery post-SBRT**	≥ 6 weeks	0.76	0.93 (0.60 – 1.45)		
	<6 weeks		Reference		
**Pathologic node status**	Positive	<0.001	2.47 (1.60 – 3.83)		
	Negative		Reference		
**Baseline CA19-9, U/mL**	≥200	0.16	1.38 (0.86 – 2.15)		
	<200		Reference		
**Age**		0.25	0.99 (0.96 – 1.01)		
**Sex**	Male	0.84	0.96 (0.62 – 1.47)		
	Female	--	Reference		
**ECOG PS**	2 or higher	0.81	1.19 (0.29 – 4.84)		
	0-1		Reference		
**Tumor Location**	Other[a]	0.14	1.39 (0.90 – 2.14)		
	Head		Reference		
**Neoadjuvant Chemotherapy Regimen**	FOLFIRINOX	0.88	0.96 (0.56 – 1.66)		
	Gemcitabine/nab-paclitaxel		Reference		
**Neoadjuvant chemotherapy duration**	≥ 4 months	0.58	0.83 (0.43 – 1.61)		
	< 4 months		Reference		
**Pathologic Complete Response**	Present	0.08	0.29 (0.07 – 1.17)		
	Absent		Reference		
**Surgical Margin**	R0	0.31	0.65 (0.28 – 1.49)		
	R1 or higher		Reference		
**Adjuvant chemotherapy**	Yes	0.23	0.77 (0.50 – 1.19)		
	No		Reference		

Overall Survival

Covariate		Univariable Cox P	Univariable Cox HR (95% CI)	Multivariable Cox P	Multivariable Cox HR (95% CI)
**Timing of Surgery post-SBRT**	≥ 6 weeks	0.21	HR 0.74 (95% CI .46 – 1.19)		
	<6 weeks		Reference		
**Pathologic node status**	Positive	<.001	2.37 (1.46 – 3.84)	<.001	2.55 (1.57 – 4.15)
	Negative		Reference		

**Baseline CA19-9, U/mL**	≥200	0.39	1.24 (0.76 – 2.01)		
	<200		Reference		
**Age**		0.28	1.02 (0.99 – 1.04)		
**Sex**	Male	0.92	1.03 (.64 – 1.64)		
	Female	--	Reference		
**ECOG PS**	2 or higher	0.094	2.71 (0.84 – 8.69)		
	0-1		Reference		
**Tumor Location**	Other[a]	0.59	1.14 (0.71 – 1.85)		
	Head		Reference		
**Neoadjuvant Chemotherapy Regimen**	FOLFIRINOX	0.99	1.00 (0.55 – 1.83)		
	Gemcitabine/nab-paclitaxel		Reference		
**Neoadjuvant chemotherapy duration**	≥ 4 months	0.12	0.60 (0.31 – 1.14)		
	< 4 months		Reference		
**Pathologic Complete Response**	Present	0.13	0.04 (0.001 – 2.38)		
	Absent		Reference		
**Surgical Margin**	R0	0.74	1.13 (0.54 – 2.37)		
	R1 or higher		Reference		
**Adjuvant chemotherapy**	Yes	0.003	0.48 (0.30 – 0.79)	0.001	0.44 (0.27 – 0.72)
	No		Reference		

Abbreviations: P, p-value; HR, hazard ratio; CI, confidence interval; SBRT, stereotactic body radiation therapy; ECOG, Eastern Cooperative Oncology Group; PS, performance status; FOLFIRINOX, folinic acid, fluorouracil, irinotecan, oxaliplatin. a Other tumor locations included pancreatic neck, body, tail, and uncinate process.

Supplemental Table S2. Sensitivity analysis excluding 4 patients who received surgery ≥15 weeks after completion of SBRT.

**Covariate**	**Univariable Cox P**	**Univariable Cox HR (95% CI)**	**Multivariable Cox P**	**Multivariable Cox HR (95% CI)**
Timing of Surgery ≥ 6 weeks post-SBRT	.038	0.53 (0.29 – 0.96)	.011	0.45 (0.24 – 0.83)
Pathologic node positivity	.033	1.91 (1.05 – 3.48)	.033	1.97 (1.06 – 3.68)
Baseline CA19-9 > 200	.03	1.97 (1.07 – 3.64)	.004	2.61 (1.37 – 4.98)
Age	.59	0.99 (0.96 – 1.03)		
Sex	.022	2.10 (1.11 – 3.96)	.044	1.94 (1.02 – 3.69)
ECOG PS (0-1 vs 2)	.76	1.36 (0.19 – 9.89)		
Tumor Location (Head vs non-Head)	.38	0.75 (0.40 – 1.42)		
Neoadjuvant Chemotherapy Regimen (FOLFIRINOX vs Gemcitabine/nab-Paclitaxel)	.25	0.62 (0.28 – 1.39)		
Neoadjuvant chemotherapy duration (>=4 or <4 months)	.98	0.99 (0.46 – 2.13)		
Presence of Pathologic Complete Response	.18	0.26 (0.035 – 1.87)		
R0 resection status	.39	1.46 (0.62 – 3.47)		

Adjuvant Chemotherapy	.38	0.81 (0.51 – 1.30)
